# Exploiting open source 3D printer architecture for laboratory robotics to automate high-throughput time-lapse imaging for analytical microbiology

**DOI:** 10.1371/journal.pone.0224878

**Published:** 2019-11-19

**Authors:** Sarah H. Needs, Tai The Diep, Stephanie P. Bull, Anton Lindley-Decaire, Partha Ray, Alexander D. Edwards

**Affiliations:** 1 Reading School of Pharmacy, University of Reading, Whiteknights, Reading, United Kingdom; 2 Centre for Electronic Imaging, The Open University, Milton Keynes, United Kingdom; 3 Department of Animal Sciences, School of Agriculture, Policy and Development, University of Reading, Reading, United Kingdom; Harvard Medical School, UNITED STATES

## Abstract

Growth in open-source hardware designs combined with the low-cost of high performance optoelectronic and robotics components has supported a resurgence of in-house custom lab equipment development. We describe a low cost (below $700), open-source, fully customizable high-throughput imaging system for analytical microbiology applications. The system comprises a Raspberry Pi camera mounted on an aluminium extrusion frame with 3D-printed joints controlled by an Arduino microcontroller running open-source Repetier Host Firmware. The camera position is controlled by simple G-code scripts supplied from a Raspberry Pi singleboard computer and allow customized time-lapse imaging of microdevices over a large imaging area. Open-source OctoPrint software allows remote access and control. This simple yet effective design allows high-throughput microbiology testing in multiple formats including formats for bacterial motility, colony growth, microtitre plates and microfluidic devices termed ‘lab-on-a-comb’ to screen the effects of different culture media components and antibiotics on bacterial growth. The open-source robot design allows customization of the size of the imaging area; the current design has an imaging area of ~420 × 300mm, which allows 29 ‘lab-on-a-comb’ devices to be imaged which is equivalent 3480 individual 1μl samples. The system can also be modified for fluorescence detection using LED and emission filters embedded on the PiCam for more sensitive detection of bacterial growth using fluorescent dyes.

## Introduction

Traditional methods of microbiological screening are time consuming, laborious, resulting in high costs for time and labour. Even within clinical microbiology labs, where a large number of samples are processed, automation remains low [1, 2]. The use of high-throughput automated systems allows increased sample processing, cost saving, and more flexibility in testing, for example screening against exogenous agents such as antibiotics for antimicrobial resistance (AMR) [1, 3]. Microbiological experiments deal with a high variety of sample formats such as soil samples and medical samples, including sputum and urine, and the protocols for analyses can be varied: from microtitre plates and agar plates, to microscopy and bacterial identification strips such as API strips. For this reason, platforms for measuring microbiological experiments tend to be highly specialised for a specific experiment, such as Biolog’s microbial identification system, or of limited use, as flexibility is difficult to achieve. While automation and partial automation of microbiological techniques may improve sample processing time, the techniques used for many experiments remain unchanged, using classic agar plate growth to culture bacteria and phenotypically study aspects such as antimicrobial resistance. Antimicrobial resistance has been identified as a global threat to human health and existing techniques to identify antimicrobial resistance remain too slow and costly to warrant a test before treatment of a patient begins. This can have a broader negative impact, through the build-up of antimicrobial resistance through over-use of antibiotics and incorrect selection leading to treatment failure and driving maintenance of resistance [4–6].

To combat this issue, improved techniques are required which provide a higher throughput and more flexible scope of analysis than traditional methods. Microfluidic techniques have been used to study bacteria phenotypes, to detect pathogenic species, and measure antimicrobial resistance at speeds approaching those required for a point of care device [7–11]. We have described a simple, low-cost microfluidic device that can be used to measure multiple antimicrobial resistance profiles of bacteria using the metabolic sensitive dye, resazurin [12] that detects bacterial growth by colour change from blue to pink. This test allows high throughput microfluidic devices, termed ‘lab-on-a-comb’ that are compatible with existing laboratory equipment, 96 well microtitre plates. These devices are made from a melt-extruded highly transparent fluorinated ethylene propylene co-polymer (FEP-Teflon®) microcapillary film (MCF) and comprises a ribbon containing an array of 10 capillaries along its length with an average diameter of 206 ± 12.6 μm. Test strips consist of a 33 mm length of MCF with each capillary internally coated with a different antibiotic of choice to test [13]. Therefore, a single well of a microtitre plate can be expanded to test for up to 10 antibiotics or 10 concentrations of a single antibiotic. While the test relies on a simple colour change and images can be analysed taking colorimetric or fluorescent images, no such reader analogous to a plate reader exists. Furthermore, to aid in the development of new technology to measure bacterial phenotypes, detailed information on bacterial growth, morphology and kinetic effects of substances is beneficial. To collect this data manually is time consuming and labour-intensive (i.e. manually taking images every hour), and therefore systems are needed that increase the automation of the analysis of microfluidic devices, and that increase sample throughput.

The development of new scientific equipment is costly and time consuming, and is repeated by laboratories all over the world, in order to achieve suitable capabilities without the high costs of proprietary scientific equipment. The time spent independently developing and re-developing these techniques hinders scientific progress globally and limits the ability of many facilities to participate in some areas of research [14]. The open-source hardware movement aims to aid rapid scientific progress by increasing the accessibility of laboratory hardware designs globally, and allowing scientists to share, utilise and improve upon hardware designs. This allows flexibility in design for specific technical requirements, giving scientists the ability to tailor their laboratory to their needs, and enables low-cost innovation in scientific methods. Growth in open source hardware designs combined with the low cost of high performance optoelectronic and robotics components has allowed a resurgence in in-house custom lab equipment development [15–19]. The use of open-source hardware in biological imaging has been successfully implemented in the use of microscopes to overcome the alternative costly and inflexible laboratory equipment, such as the use of a Raspberry Pi computer and camera alongside Arduino-based optical and thermal control circuits for microscopic monitoring of model organisms [20]. The Raspberry Pi camera has likewise been combined with an elegant x-y-z micron precision stage that exploits the flexibility of a 3D printed polylactic acid plastic frame to deliver a versatile, compact, 3D printed open source digital microscope [21].

The concept of an open-source, low cost 3D printer capable of printing the majority of its own components was proposed by Adrian Bowyer [22], and is known as the RepRap movement (replicating rapid prototype). The RepRap movement led to a massive expansion of accessible, low-cost high-performance 3D printers. This open-source hardware has been utilised as an affordable method of 3D printing laboratory equipment by scientists in various fields [23–25]. We have exploited RepRap 3D printer architecture to develop a low cost (below $700), open-source, fully customizable robotic high-throughput imaging system for analytical microbiology applications called POLIR (raspberry Pi camera Open-source Laboratory Imaging Robot). The frame and x-y-z motion is designed around simple v-slot aluminium extrusion widely available across the world and allowing simple customisation. This system is capable of taking time-resolved images of large panels of samples of different microbiological based assays that use petri dishes, microtitre plates and microfluidic devices, with time intervals of as little as seconds, and total experimental times of up to days. Using existing open-source 3D printer designs published under creative commons licences, the frame was adapted to hold a Raspberry Pi camera attached to a z-linked actuator to adjust z-height, replacing the extrusion head. All resources developed are available under open-source licenses and are deposited in GitLab.

While several instruments already exist for use in specific microbiological experiments they are usually designed for a single purpose, i.e. they can only study one petri dish at a time [17] or microtitre (MTP) plates of a certain size but not both [26]. Furthermore, these may only be used for endpoint experiments and not suitable for using in experiments that aim to determine kinetic parameters. The POLIR system is designed to take images over an area of 300 × 420 mm, with larger frames possible by simply using a longer aluminium extrusion frame. Due to the simple design, many different microbiological assays using colorimetric or visible detection can be measured in a single experiment. The simple adaptation by addition of high-power single-wavelength coloured LEDs and emission filters permits fluorescent imaging. The z-axis allows images of samples at different heights to be collected without modifying zoom or camera focus, simplifying experiments where different culture formats are run in parallel (e.g. agar plate plus microfluidic device). This system sits in a walk-in 37°C incubator room to maintain temperature, however, it is possible to add a heated enclosure around the imaging area if an incubator room is not available.

While other equipment exist for monitoring bacterial growth over time such as heated plate readers that take measurements over time, these systems can only measure one plate at a time, a total of 96 samples. Other analytical microbiology systems, such as Biolog and the Omnilog reader, support bacterial cell phenotyping using preloaded 96 well plates and a reader that allows kinetic analysis of single plates or up to 50 MTPs (4800 samples), monitored every 15 minutes and includes software for data analysis [26]. While these systems already exist and prove the value of kinetic microbial growth analysis, the instrumentation is not widely available to all labs and are restricted to specific proprietary plates and experimental formats. The POLIR platform can monitor 10 plates (960 samples), and a larger frame would easily permit more plates to be monitored for only the cost of longer aluminium extrusion.

A key application for flexible time-resolved automated imaging is for optimising microbial detection and antimicrobial resistance measurement, important for a wide range of applications including healthcare (e.g. infection, pharmaceutical manufacturing) and environmental (e.g. antimicrobial resistance spread from agricultural use of antibiotics). Development of rapid AMR tests would ideally measure bacterial resistance to antibiotics directly from the sample. In many cases a change in sample matrix can affect both microbial detection and antibiotic activity. For example, for use in the dairy industry it would be ideal to analyse milk samples from dairy cows with mastitis for antibiotic resistance; however, milk powerfully scatters light and is therefore likely to strongly affect colour-based or fluorescent growth detection. Furthermore, milk components may potentially absorb antibiotics (reducing activity) but conversely may in some cases increase microbial sensitivity to antibiotic action, if antimicrobial components are present within milk. It consequently becomes important to study the effect of sample matrix on microbial growth. We therefore used this example to explore whether high throughput analytical microbiology with POLIR can accelerate the analysis of sample matrix interference on antimicrobial susceptibility testing in both conventional and microfluidic test formats.

## Materials and methods

### Imaging robot design concept and assembly

We assembled a Core X-Y RepRap 3D printed frame with a moving distance of 300 × 300 × 130 mm adapting the D-bot 3D printer design [6]. The system is controlled by the open-source software OctoPi running an OctoPrint server. OctoPrint is commonly used to connect remotely to 3D printers and often utilises a webcam or PiCam to monitor printing progress. The 3D printing extruder was replaced by a Raspberry Pi singleboard computer and PiCam module mounted on a vertical linear actuator (built to an OpenBuilds design [27]) to adjust the z-height, allowing camera focus. The PiCam lens was rotated anticlockwise to focus closer than the supplied infinity focus, allowing a range of fields of view and working distances. We selected a working distance of 80 mm that gave a field of view of 96.5 × 72.5 mm. OctoPrint controls the position of the camera using custom G-code, by supplying G-code from the Raspberry Pi to the Arduino mega board used to control the x-y-z stepper motors. The Arduino board firmware was open-source Repetier software that interprets the G-code driving stepper motors via a RepRap RAMPS 1.4 shield. To take images a Python script configured to acquire the required PiCam image settings (e.g. image exposure, resolution, time) is triggered as an executable shell script via serial command embedded within the G-code and executed by the OctoPrint G-code system command plugin. The images are then stored onboard the Raspberry Pi SD card and can be accessed remotely by file transfer protocol (FTP).

The positional accuracy of the POLIR was tested using G-code to replicate an experiment moving over thirteen overlapping areas of the white LED. An USAF 1951 resolution target was included in one of the areas, and the image sequence repeated every 10 minutes, with each cycle moving over the thirteen areas again over 4–16 hours. The robot was either homed or not between each run of thirteen moves. The depth of focus was determined by sequentially imaging the USAF 1951 resolution target at multiple z heights, either on the surface of the LED light box, or on the surface of an agar petri dish (approximately 4 mm deep agar medium). In all cases, the location of a target line was recorded to determine positional accuracy, and the smallest line pairs that could be resolved clearly was recorded to indicate the image resolution.

The designs, hardware links and software links for the POLIR have been deposited in GitLAB (https://gitlab.com/AlEdwards/polir) [28]. The design for the POLIR was based on open-source designs for 3D printers that can be found at [29] along with the design for the linear actuator from OpenBuilds [27].

### Microbiology methods

A soft agar motility assay was performed to determine phenotypes of bacteria. Reference strains of *E*. *coli* ATCC 25922 and *Staphylococcus aureus* ATCC 12600 were used (LGC group, Middlesex, UK). The bacterial strains were routinely cultivated on lysogeny broth (LB) agar (Sigma-Aldrich) at 37°C. Agar at 0.8% in LB broth was autoclaved and, once cooled to 50°C, supplemented with a final concentration of 0.1 mg/mL Triphenyltetrazolium chloride (TTC, Sigma Aldrich) and 1 mL poured in each well of a 12 well sterile plate. Bacteria was grown overnight in LB broth and normalised to 0.5 McFarland standard and diluted to 1 in 10 000. A 1 μL loop was used to stab the agar to three quarters its depth and the bacteria was incubated overnight at 37°C and images collected every hour.

To study colony growth on agar plates, bacteria were grown overnight in LB media and normalised to 0.5 McFarland standard. The samples were further diluted to give 10^7^, 10^6^, 10^5^, and 10^4^ CFU/mL and 10 μL was spread over LB agar (Sigma Aldrich) plate supplemented with 0.1 mg/mL TTC (Sigma Aldrich) followed by overnight incubation and colony counting to determine CFU/mL.

For microfluidic assays in microcapillary film (MCF), each comb consisted of a custom 3D printed MCF holder containing a row of 33 mm long Lab-on-a-Stick [30] test strips at 9 mm pitch to match microtiter plates (S1 Fig). The fluorinated ethylene propylene MCF was manufactured by melt-extrusion by Lamina Dielectrics Ltd (Billingshurst, West Sussex, UK) from a highly transparent fluorinated ethylene propylene co-polymer (FEP-Teflon®) and comprises a ribbon containing an array of 10 capillaries along its length with an average diameter of 206 ± 12.6 μm and external dimensions of 4.5 ± 0.10 mm wide by 0.6 ± 0.05 mm thick. For each batch, 1 m MCF lengths were internally coated by incubation with a 5 mg/mL solution of polyvinyl alcohol (PVOH) in water (MW 146,000–186,000, >99% hydrolysed, Sigma-Aldrich, UK) at room temperature for a minimum of 2h [31]. Coated strips were washed with 5 ml of PBS with 0.5% Tween 20 (Sigma-Aldrich, UK) to remove residual PVOH, and dried with compressed air at 2 bar pressure for 20 minutes. For antimicrobial resistance tests 1 m lengths of MCF were loaded with duplicate capillaries of gentamicin or ampicillin (both at 10 mg/mL dissolved in water). The antibiotic was removed using a vacuum pump leaving behind a thin film of reagent[13].

Bacterial inoculums were prepared according to British Society for Antimicrobial Chemotherapy (BSAC) standard for antimicrobial susceptibility tests[32, 33]. Briefly, an overnight culture was adjusted to a 0.5 McFarland, diluted to the indicated CFU/mL concentration in Mueller-Hinton broth (Sigma Aldrich) supplemented with 0.25 mg/mL resazurin sodium salt powder (Sigma-Aldrich, UK). MCF was cut into 33 mm test strips that were dipped using a custom 3D printed holder into the bacterial inoculum in a microtiter plate (S1 Fig) and incubated overnight at 37°C. Test strips were imaged by the POLIR in an incubator room at 37°C, with a white background illumination to record colour change of the resazurin dye indicator; a change from blue to pink indicating resazurin conversion following bacterial growth. To study the effect of milk on bacterial growth, sterile milk (Tesco, UK) was added at the indicated concentration and diluted with Mueller-Hinton Broth such that the Mueller-Hinton was always at 1X. To determine if POLIR could be used to measure growth kinetics in MTP, parallel bacterial growth kinetics through colour change was prepared, and recorded in MTP alongside MCF. For fluorescence measurements, resazurin was used at a final concentration of 60 μg/mL.

Mastitis milk samples were collected from the University of Reading Farm. Serial ten-fold dilutions in peptone water (Sigma) were directly streaked on gram-positive and gram-negative chromagar plates (CHROMagar™ Mastitis). Plates were incubated at 37°C overnight and bacteria were identified based on the colony colour. Isolates were grown overnight in LB media and diluted 1:200 followed by 10-fold dilutions and growth was monitored fluorescently by the POLIR for 12 h in the presence and absence of gentamicin and ampicillin (loaded at 5 mg/mL in water).

The ends of the MCF were sealed using a custom 3D printed cap filled with silicone grease to avoid sample evaporation. Analysis of MCF microfluidic devices was performed in MatLab and analysis of microtiter plates used ImageJ.

## Results

The POLIR was based on a 3D printer configuration known as CoreXY [34]. This open-source 3-axis robot was developed to maximise positional accuracy whilst minimizing cost for rapid and programmable x-y-z positioning of an extruder for fused filament deposition 3D printing, following the RepRap principles of 3D printed components allowing self-replication. To address the microbiology laboratory's need for cost-effective, open-source, programmable x-y-z positioning of a lab imaging camera, we selected this design as a basis for developing an open-source laboratory imaging robot.

**Fig 1:**
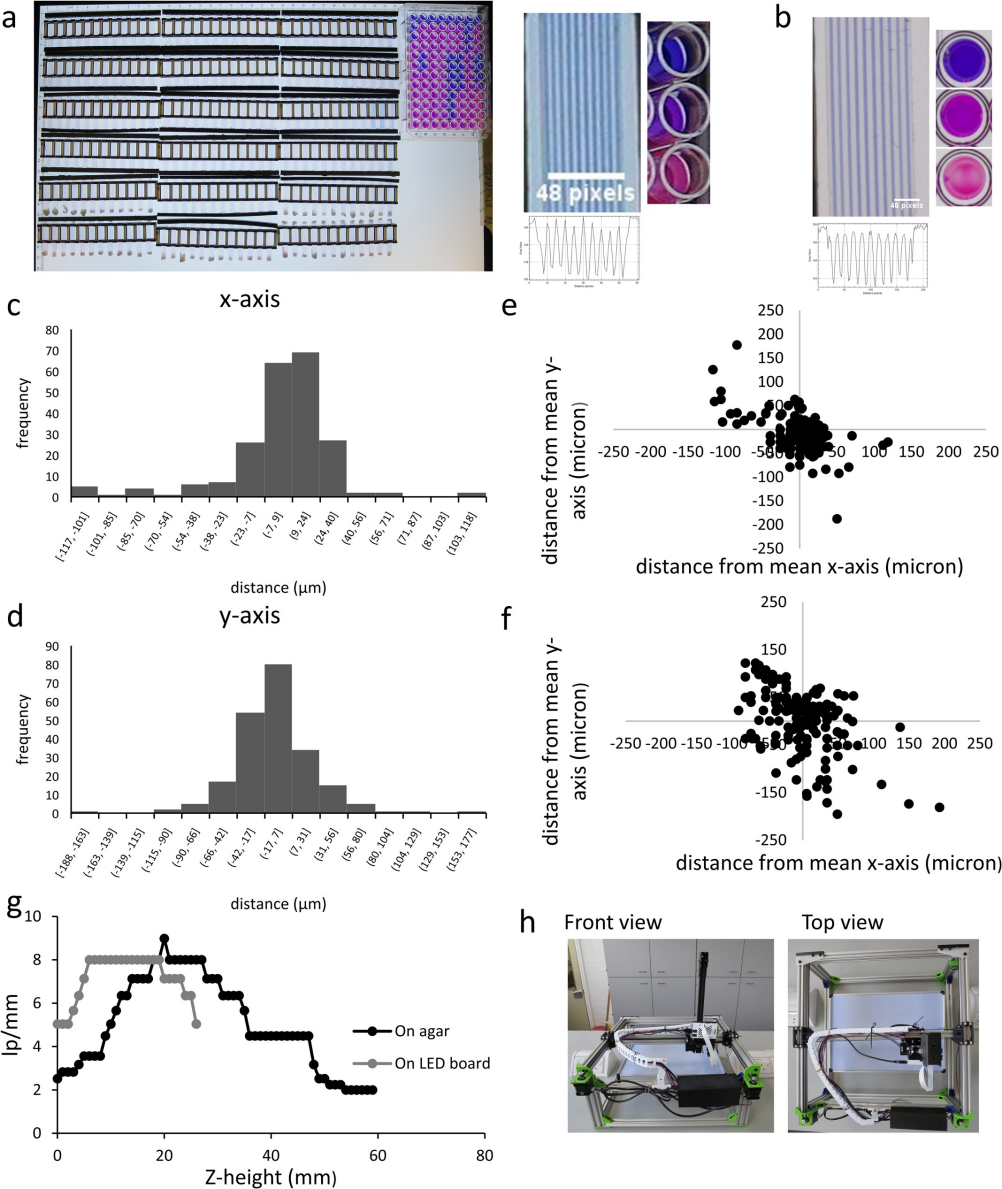
POLIR architecture, positional accuracy, and resolution. A3 imaging size of POLIR filled with 2160 individual capillaries and a 96 well plate. The area was imaged using a Canon EOS 1300D with ES-F f/2.8 Macro Lens (a) or individual images using the POLIR (b). Scale bar indicates 48 pixels. Histogram of the deviation of repeated measurements from the mean for the x-axis (a) and y-axis (b). Scatter diagrams showing the x and y positions in relation to the mean when there is no homing (c) and with homing every 13 image acquisitions (d). (e) line pairs/mm in relation to the z-height of the camera axis for objects flat on the white light LED board and on agar plates. (f) Image of POLIR from front view and top view. All files needed to build the POLIR have been deposited on GitLAb licenced under the MIT license. (g) Number of line pairs/mm from USAF 1915 resolution target seen at increasing z-height. (h) Photograph showing the front and top view of the POLIR.

This robot was designed to be able to take detailed time-lapse images and record kinetic data for large number of samples whilst decreasing the amount of hands on time for the experimenter, who would otherwise need to make repeated measurements (e.g. in a plate reader or with camera). To be able to cope with large number of samples a moveable camera is beneficial. Using 18 ‘lab-on-a-comb’ devices and a 96 well microtiter plate, images of the whole experimental area were taken using a Canon EOS 1300D with ES-F f/2.8 Macro Lens costing approximately $700, which is the entire cost for the POLIR system. Using a fixed camera for the entire imaging area of the POLIR does not allow for high resolution images. Using the Canon system, each capillary width of our microfluidic device only accounts for 1.5 pixels (Fig 1A). Using a moveable camera allows a shorter focus with each capillary made up of 6 pixels (Fig 1B). Static cameras would also be a problem for imaging multiple formats of different heights. Microtitre plates need to be imaged from above each well or the sides of the well begin to obscure the sample to be measured (Fig 1A and 1B).

A key aspect of increasing the information obtained per experiment with decreased hands on time for the experimenter relies on automated image analysis. Simple image analysis automation such as MatLab scripts are made easier when each image is in the same place every time-point, especially with microfluidic devices with a small measurement region of interest. To determine the suitability of POLIR for automated microfluidic device image analysis, positional accuracy was measured. The POLIR was run through a full experimental setup for 4–16 h imaging every 10 minutes including a USAF 1915 resolution target. For experiments that were homed in the x, y and z axis once at the beginning of the experiments we found that the average distance in movement frame to frame from the mean for x and y-axis is 20 ± 1.36 and 26 ± 1.76 μm respectively. The majority (98%) of data points fell within 100 μm of the mean (Fig 1C–1E), therefore allowing MCF with a capillary diameter of 200 μm to be imaged successfully and followed by automated image analysis. Experiments that were homed at the beginning of each image (every 10 minutes) had a greater degree of movement (Fig 1F). Homing at the start of each image acquisition resulted in an average movement of 35 and 29 μm from the mean. Finally, to confirm resolution and depth of focus, the USAF 1951 resolution target was used to identify z-height for maximal resolution (Fig 1G). These experiments used a working distance of 80 mm and field of view of 90 mm, which gave over 10 mm depth of focus with maximal resolution of 8 line pairs per mm corresponding to 62 μm line width, 4× smaller than the 200 μm capillaries within MCF. All subsequent experiments presented here used this optical setup, however the PiCam is versatile and simple adjustment of the stock lens allows closer working distance with higher magnification and resolution, since the camera is attached to a z-linked actuator it is also possible to image experimental setups with different heights to maintain focus (Fig 1H).

**Fig 2:**
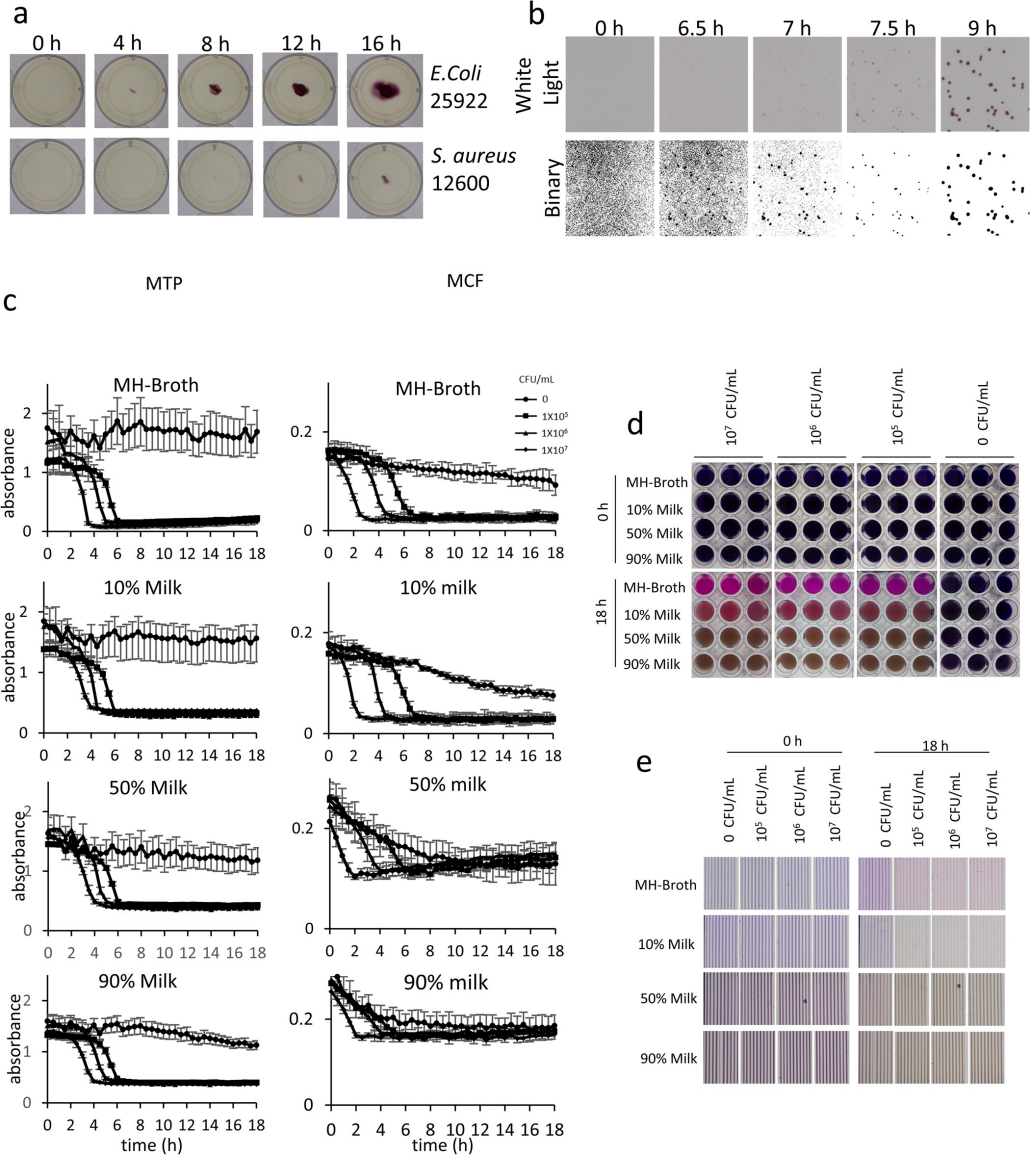
Demonstration of different experimental formats performed using POLIR using agar plates, microtitre plates and microcapillary film. (a) Motility assay using motile *E*. *coli* 25922 and non-motile *S*. *aureus* 12600. (b) Detection of *E*. *coli* colonies on LB agar supplemented with 0.1 mg/mL TTC showing both the raw and binary images. (c) Effect of milk concentration on *E*. *coli* growth and detection within MCF, error bars indicate ± SD of 3 replicate wells for MTP or 10 capillaries for MCF. (d) Images of MTP wells at start and endpoint of data shown in Fig 2C. (e) Images of MCF at start and endpoint of data shown in Fig 2C.

Having established the positional accuracy of POLIR, we wished to test the system for recording microbial movement by imaging motility assays. Motility assays can be used to determine phenotypes of bacteria [35]. Conventional motility assays are interpreted as an endpoint after overnight incubation. The POLIR allows detailed measurement of kinetic motility analysis (S1 and S2 Files), with the difference between motile *E*. *coli* and non-motile *S*. *aureus* being clear as early as 7.5 h (Fig 2A). Time-lapse imaging increases the quantity and quality of bacterial motility data and would permit detailed analysis of the effect of exogenous stimuli on growth and motility, i.e. delayed or increased speed of motility. This experiment was performed in a single 12-well plate; if the full imaging area of the POLIR was used, this would allow 10 × 12 well plates giving a total of 120 conditions that could be screened simultaneously. The POLIR can monitor colony growth to determine speed of growth and colony morphology. *E*. *coli* 25922 was grown on LB agar supplemented with the insoluble formazan dye, TTC, used to increase bacterial colony contrast against the white light brightfield format (Fig 2B and S3 File). Simple image manipulation to make the images binary aids in morphology mapping and allows colonies to be detected earlier and much smaller giving more detailed information on size, shape and growth over time.

The rise in antimicrobial resistance has been partly exacerbated by the injudicious of antibiotics. This can be attributed to traditional techniques proving too slow to provide a diagnosis before treatment is prescribed [6].The possibility of a direct POC antimicrobial test is therefore appealing [36–38]. One such test that could be used to improve selection of the right antibiotic and minimise excessive use of antibiotics is against bovine mastitis. Mastitis is the infection of the mammary gland and treatment involves antibiotics. However, due to the spread of antibiotic resistance many infections are not simple to treat [39]. Microfluidic devices are well-placed for use in diagnostic testing due to their small size making them more portable than other techniques. We have explored the use of MCF as a diagnostic for antimicrobial resistance in dairy cow mastitis. One of the major pathogens that causes mastitis in dairy cattle is *E*. *coli* [40]. To be able to directly test AMR in milk samples the effect of milk as a matrix for bacterial growth was needed. Bacterial growth was measured using the metabolic sensitive dye, resazurin. By using the POLIR we can test side by side the MCF device MTP with kinetic data of *E*. *coli* 25922 growth in increasing concentrations of milk.

This gives valuable information, if only the beginning and endpoint results were taken it would be unclear whether concentrations of milk higher than 50% have differences dependent on bacterial count concentration as the no bacterial control still converted the resazurin but at a much slower rate (Fig 2C). Milk concentration also affects the absorbance measurements in MCF to a greater degree than in MTP indicating samples would have to be diluted to determine bacterial growth in MCF. This is due to the decreased pathlength in the MCF compared to the MTP which decreases the colour intensity. At the same time, milk contains more light scattering particles making the sample appear opaque [41]. This decreases the range between the starting blue and endpoint pink. There is also a general decrease in absorbance in MCF with no bacteria which is exacerbated by increasing milk concentrations. While a small amount of resazurin may be converted to resorufin in high milk concentrations, this is not seen in MTP due to the longer pathlength, the overall colour remains blue as it has more intensity than in the MCF.

Previous experiments using MCF microfluidic devices have been restricted to manually taking images, due to its non-standard format, restricting the number of data points and time of monitoring from around 7 data points over 7 h. The POLIR allows for monitoring over much longer time periods and depending on the need, imaging as often as every 10 s in a static position or every 15 minutes using the entire imaging area. This allows increased accuracy when modelling growth kinetics.

**Fig 3:**
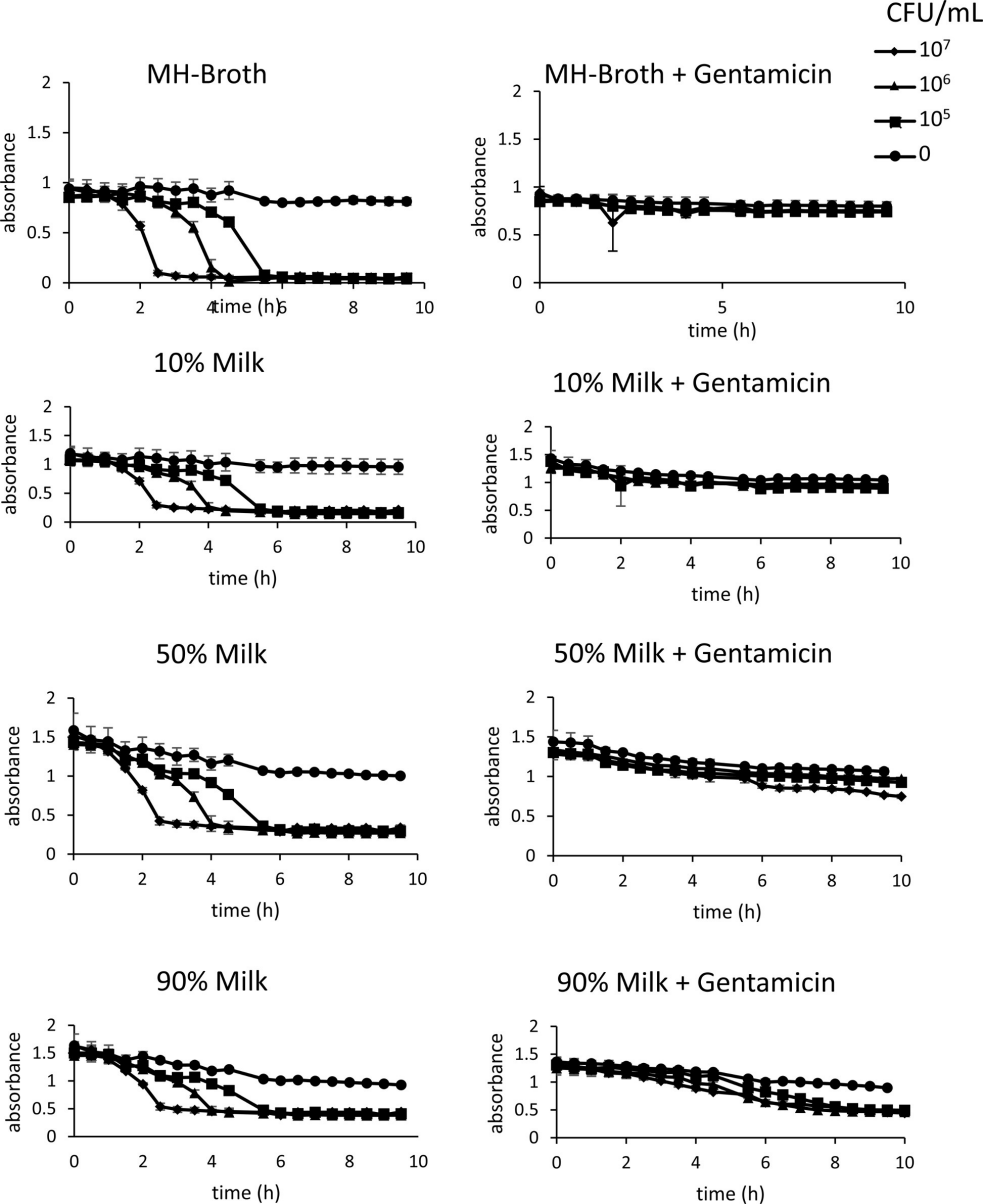
POLIR can be used to screen antibiotic resistance of bacteria. Growth curves of *E*.*coli* 25922 grown in MH broth or 10, 50 and 90% sterilised milk diluted in MH broth without and with 5 μg/mL gentamicin in MTP. Error bars indicate ± SD of triplicate wells in microtitre plate.

Different sample matrixes can affect antibiotic function as well as dye conversion. The effect of increasing concentration of milk on bacterial (*E*. *coli*) resistance a single concentration of gentamicin milk was tested using the POLIR in microtitre plate. While there was no visible effect on resistance profile in 10% milk when compared to Mueller-Hinton broth, concentrations higher than 50% showed colour change in conditions even in the presence of gentamicin. This was reflected in MCF indicting that any direct testing of AMR in milk would have to be diluted to a minimum of 1 in 10 before testing (Fig 3).

**Fig 4:**
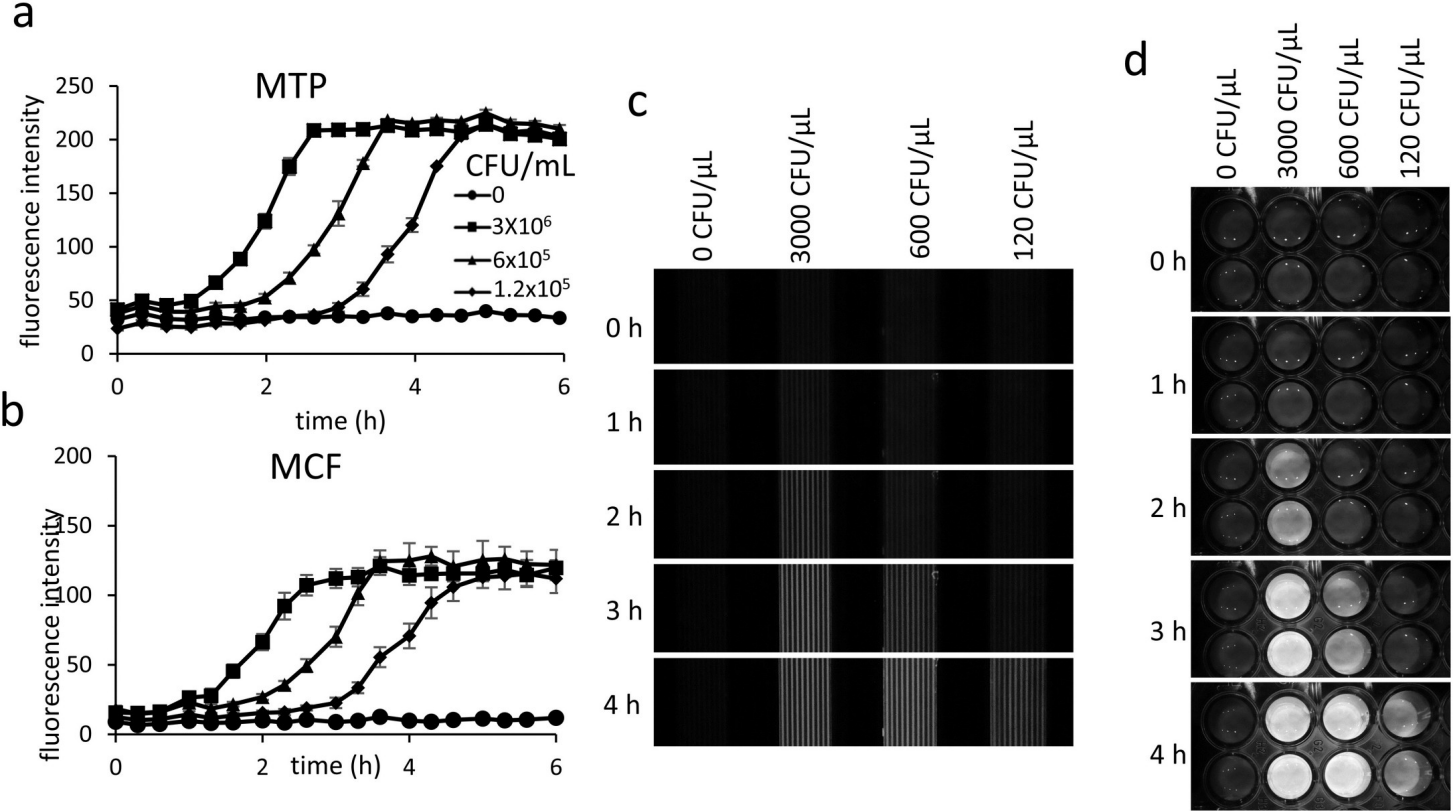
Adaptation of POLIR for fluorescence measurements. Growth curves of *E*. *coli* 25922 detected by fluorescence increase in resazurin metabolism measured in microtitre plates (a) and micro capillary film (b) Error bars indicate ± SD of duplicate wells in microtitre plates or 10 capillaries in microcapillary film. (c) Images captured from POLIR of microtitre plate and (d) microcapillary film.

To increase the applications of the POLIR the system can be modified for fluorescence detection using low-cost LEDs and coloured glass emission filters embedded on the PiCam for more sensitive detection of bacterial growth using fluorescent dyes (Fig 4). Metabolism of resazurin changes colour from blur to pink but also from non-fluorescent to highly red fluorescence compound, resorufin. Fluorescent detection of resazurin also allows a smaller amount of dye to be used in the microcapillary film going from 250 μg/mL to 60 μg/mL while still being visible (S2 Fig). Fluorescence detection of resazurin conversion is more sensitive than absorbance measurements and increases speed in detection of *E*. *coli* 25922 by approximately 2 h. The current design only allows a single fluorescence wavelength to be measured at one time, but the system is flexible enough to change the LEDs and filters to the desired wavelength of choice.

The POLIR was used to screen 24 bovine mastitis samples against gentamicin and ampicillin. Ampicillin and gentamicin were loaded into duplicate capillaries and tested against four dilutions of bacteria and growth was detected by fluorescent intensity of resorufin. Isolates were tested with duplicate strips with four ten-fold dilutions. Three of the isolates were tested using overnight liquid culture or resuspension of colonies from a fresh agar plate. A total of 216 test strips corresponding to 2160 individual capillaries were measured every 30 minutes for 12 h (S3 Fig). Collecting kinetic data of multiple dilutions allowed the doubling times of the isolates to be calculated while simultaneously measuring susceptibility to ampicillin and gentamicin (Table 1). The different isolates had different effects on resazurin metabolism (S3 Fig). Resazurin has been proposed for high-throughput screening for antimicrobial assays due to it’s metabolism into the highly fluorescent resorufin [42–44], however, resorufin can be further metabolised into the non-fluorescent dihydroresourfin [42] leading to a transient fluorescent signal in some systems. Transient fluorescent signals can be seen in a number of the mastitis isolates (S3 Fig) and is not-related to generation time. Using end-point measurements would lead to misinterpretation of results.

**Table 1:** Doubling time and resistance/susceptibility profile of bacteria isolated from bovine mastitis samples.

Isolate Number	Isolate Code	Doubling Time (minutes)	Gentamicin	Ampicillin
1	19.02 MRF GN	23	S	S
2	19.02 MRF GPC blue	33	S	S
3	19.02 MRF GNC white	*nd*	*nd*	*nd*
4	19.05 MRF GPC blue	19	S	S
5	19.16 MRF *S*. *uberis*	37	S	S
6	19.13 MRF Proteus	31	S	S
7	19.10 MRF PSA	29	S	S
8	19.08 MRF SA	26	S	S
9	19.10 MRF *S*. *uberis* (13)	29	S	S
10	19.06 MRF GPC blue	26	S	S
11	19.16 MRF EC	15	S	S
12	19.15 MRF SA	26	S	S
13	19.13 MRF SA	*nd*	S	S
14	19.11 MRF	*nd*	*nd*	*nd*
15	19.02 MRF LC+	18	S	S
16	19.02 MRF L-	25	S	S
17	19.12 MRF	*nd*	*nd*	*nd*
18	19.10 MRF S. uberis	33	S	S
19	19.17 MRF EC	13	S	S
20	19.17 MRF	*nd*	*nd*	*nd*
21	19.14 MRF S. uberis	*nd*	*nd*	*nd*
22	19.14 MRF KLEB	49	S	R
23	19.14 MRF SA	*nd*	*nd*	*nd*
*24*	*E*. *coli* ATCC 25922	16	S	S

## Discussion

Open-source laboratory hardware has recently drawn a large community of researchers, with new designs for different applications frequently emerging.

A significant advantage of the POLIR over traditional methods of imaging, is the remote access of the system, which can be monitored and controlled via a computer or smartphone device, giving the researcher flexibility in when they can run experiments and check the progress of an experiment in real time, reducing the need for hands-on time imaging the experiment. Compared to relatively common automated plate readers, the POLIR allows kinetic data collection for multiple plates simultaneously as well as other microbiology-based formats, giving high-throughput, flexible analysis (Table 2). The precise movements of the POLIR results in uniform images over time. Experimental analysis is often one of the bottlenecks in research labs dealing with image analysis. One important aspect of analysis automation is to keep images in the same location every time. When taking images manually using a camera it is nearly impossible to get the frame exactly right. However, using the POLIR most images are within 50 μm of the mean and when looking at peak heights a small variation control is an easy addition in analysis programmes. This is especially important when imaging small devices, such as MCF where each capillary is only 200 μm wide. Automation allows large number of images to be analysed in the time it takes to analyse a single image manually.

**Table 2:** Summary of POLIR benefits over microplate reader or static camera.

Characteristic	POLIR	Microplate reader	Static camera
Throughput	High: number of plates or MCF only limited by size of frame chosen	Low: one plate at a time with no option for other formats	High: number of plates limited by resolution and distance of camera
Image analysis	Can be automated	Highly automated	Can be automated
Resolution	High	NA	Low: obscured wells at edge of image also lowers quality
Flexibility	High: can image anything	Low: only used for microplate assays	High: can image anything
Hands-on time	Low: camera is automated and can be controlled remotely	Medium: each plate must be placed into the reader for each time-point unless the temperature can be controlled	Low: camera can be set up for time-lapse
Data quality	High quality image for each capillary, well or colony	High quality data for each well	Low quality or no data for MCF capillaries and decreasing quality for microplate wells towards the edge of the image

This locational accuracy allows for rapid image analysis as positional inputs for colorimetric of fluorescent data do not need to be manually assigned for each image, even laboratories not using programming for image analysis such as MatLab or Python can take advantage of ImageJ region of interest manager which is routinely used in life sciences laboratories.

The main limitations of POLIR are the need for specific laboratory conditions. The current design for the POLIR does not include in a built-in incubator and experiments are performed in a walk-in incubator room which is not available to all laboratories. Incubators can be easily adapted to work alongside POLIR, either within the frame using heating elements and a clear lid, or by placing POLIR inside an incubator.

The open-source robot design allows customization of the size of the imaging area; with the current design, with an imaging area of ~300 × 420 mm, if custom MCF holders with a 6 mm pitch are used a total of 432 MCF strips can be imaged giving a total of 4320 individual 1 μl samples. The current configuration 3280 × 2464 pixel image has a field of view of 96.5 × 72.5 mm with 29 μm/pixel, providing a total imaging resolution exceeding 14,000 × 9800 pixels over an A3 light box. This level of magnification is useful as it provides enough resolution to see individual microcapillaries of MCF with a diameter of 200 μm while allowing at least 4 strips to be in view, decreasing the number of total images need to be taken per experiment. It also allows multiple wells to be viewed when imaging microtitre plates. Higher magnification imaging can be easily achieved by simple modification of the PiCam optics that allows closer focusing. While the size of POLIR can be adjusted for any experimental area, the time taken to image each location will increase with experimental area, and therefore the image frequency interval for each location will increase.

In conclusion, the POLIR is a low-cost imaging tool that allows the recording of colorimetric and fluorescent based microbiology experiments. The POLIR increases throughput and data density of experiments with no increased hands-on time of the experimenter. The simplicity of the design makes the POLIR amenable to adaptation for different experimental needs and the open-source nature allows it to be easily accessed and customized by other researchers.

## Supporting information

S1 Fig Microcapillary film experimental setup. Custom 3D printed holders with 9 mm pitch hold the 33 mm microcapillary film (a). The holders are compatible with 96 well microtitre plates allowing each well to be expanded into 10 capillary tests (b). The ends of the microcapillary film are sealed with a 3D printed cap filled with silicone grease to stop evaporation (c). Multiple test strips in holders are placed on the POLIR to measure either absorbance or fluorescence (d).
(TIF)

S2 Fig Effect of resazurin dye concentration on speed of detection. *E*.*coli* 25922 was grown in Mueller-Hinton broth supplemented with the indicated concentration of resazurin. Fluorescence intensity is normalised to the starting intensity. Mean indicates average of 10 microcapillaries. Error bars indicate ± SEM.
(TIF)

S3 Fig Fluorescence conversion of resazurin by isolates form dairy cattle mastitis samples with and without gentamicin and ampicillin. Negative indicates no antibiotic, ampicillin and gentamicin (loaded at 10 mg/mL). Isolates were grown overnight and diluted 1:200 diluted in Mueller-Hinton Broth with 0.06 mg/mL resazurin followed by 10-fold dilutions. Images were taken every 30 minutes. Negative indicates average of 6 capillaries, ampicillin and gentamicin are the average of 2 capillaries.
(TIF)

S1 File Timelapse video of *E*. *coli* motility test presented in Fig 2A. (ZIP)

S2 File Timelapse video of *S*. *aureus* motility test presented in Fig 2A. (ZIP)

S3 File Timelapse video of data presented in Fig 2B. (ZIP)

S4 File Timelapse video of data presented in Fig 4B. (ZIP)
