# Supplementary figures and images for: Exploiting open source 3D printer architecture for laboratory robotics to automate high-throughput time-lapse imaging for analytical microbiology
Source: PLoS One. 2019 Nov 19;14(11):e0224878. doi: 10.1371/journal.pone.0224878 (PMC6863568; doi:10.1371/journal.pone.0224878)

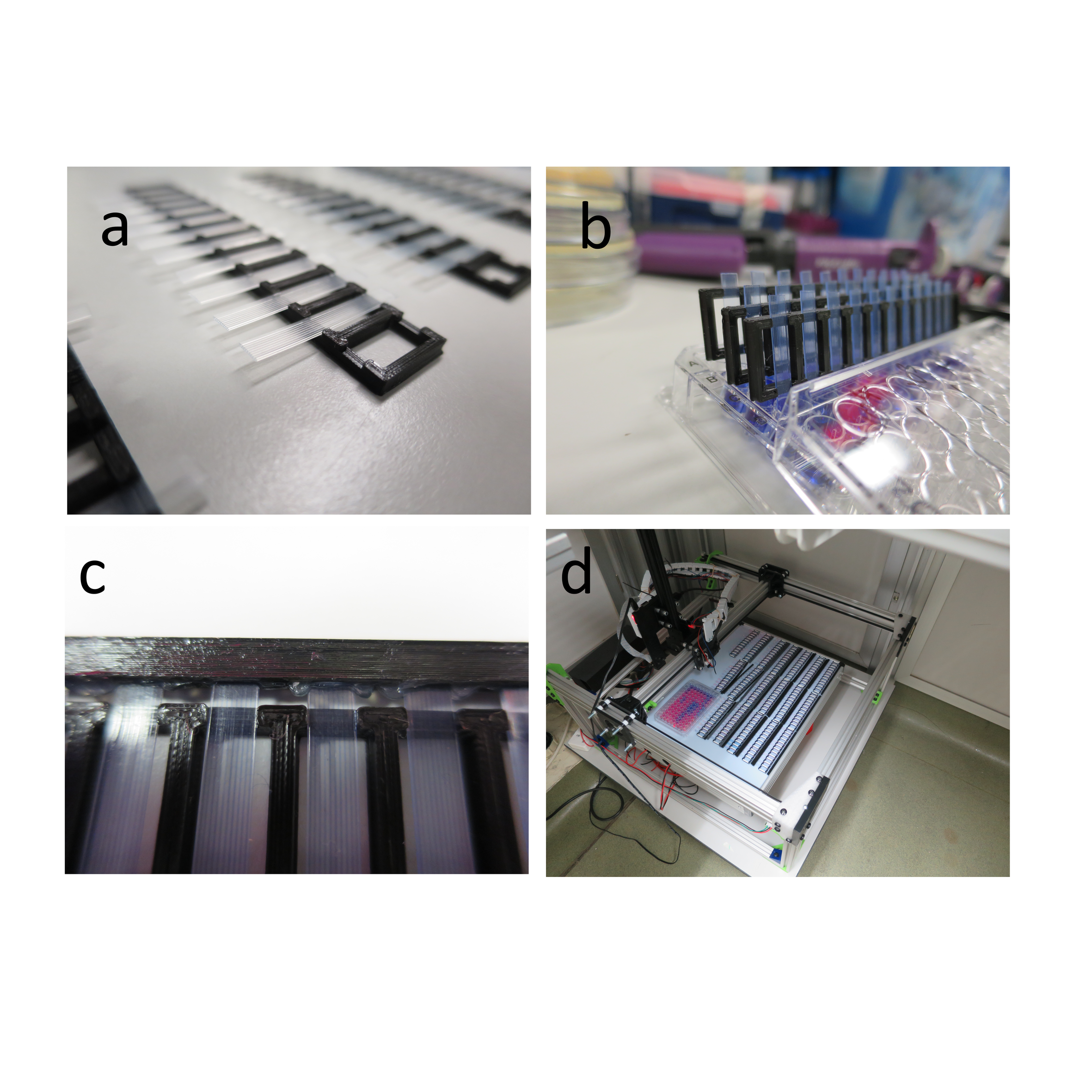

Supplement: S1 Fig — Custom 3D printed holders with 9 mm pitch hold the 33 mm microcapillary film (a). The holders are compatible with 96 well microtitre plates allowing each well to be expanded into 10 capillary tests (b). The ends of the microcapillary film are sealed with a 3D printed cap filled with silicone grease to stop evaporation (c). Multiple test strips in holders are placed on the POLIR to measure either absorbance or fluorescence (d). (TIF) [file pone.0224878.s001.tif]

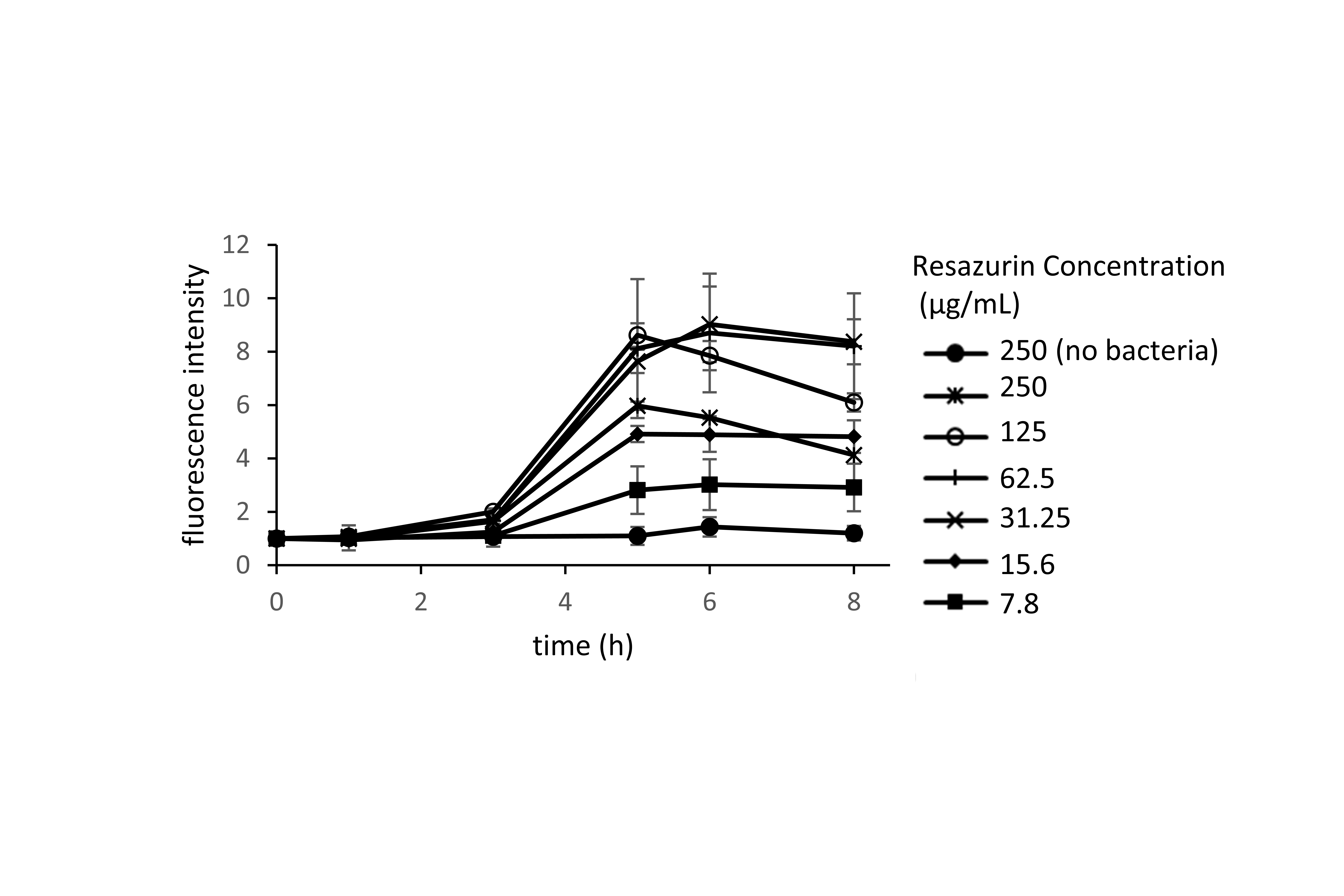

Supplement: S2 Fig — E.coli 25922 was grown in Mueller-Hinton broth supplemented with the indicated concentration of resazurin. Fluorescence intensity is normalised to the starting intensity. Mean indicates average of 10 microcapillaries. Error bars indicate ± SEM. (TIF) [file pone.0224878.s002.tif]
